# State-of-the-Art Transcranial Sonography for Neuropsychiatric and Dementia Disorders: A Narrative Review

**DOI:** 10.7759/cureus.89278

**Published:** 2025-08-03

**Authors:** Roberto P Santos, Rita de Cássia L Fernandes

**Affiliations:** 1 Service of Neurology, Hospital Universitário Clementino Fraga Filho, Universidade Federal do Rio de Janeiro, Rio de Janeiro, BRA

**Keywords:** alzheimer’s dementia, dementia, dementia research, mood disorders, transcranial doppler ultrasound, transcranial usg, ultrasonography, ultrasound, vascular dementia

## Abstract

Transcranial sonography (TCS) is widely acknowledged as a frontline imaging tool in movement disorder practice, particularly for separating idiopathic Parkinson’s disease from its many mimics. In recent years, however, investigators have extended its reach, showing that the same portable probe can also capture structural and hemodynamic signatures of neuropsychiatric disorders and the major dementia syndromes.

Across neuropsychiatry, a dim (“hypoechoic”) median raphe emerges as the sonographic hallmark of serotonergic imbalance: it recurs in major depressive disorder, bipolar depression, and panic disorder, predicts better response to selective serotonin reuptake inhibitors, and even foreshadows post-stroke depression. Conversely, a bright, enlarged substantia nigra, the classic Parkinson marker, also surfaces in a subset of depressed patients and in some antipsychotic-treated individuals, implying latent dopaminergic stress unmasked by medication.

In dementia research, TCS reliably tracks structural and hemodynamic changes that mirror, or sometimes precede, findings on MRI. Third-ventricle expansion correlates with falling Mini-Mental State Examination and Montreal Cognitive Assessment (MoCA) scores, while simple linear indices of the mesial temporal lobe (the medial temporal lobe-to-choroidal fissure ratio, the medial temporal lobe atrophy score in sonography (MTA-S), and the ventricle enlargement score in sonography (VES-S)) separate Alzheimer’s disease from normal aging with MRI-like accuracy, but none of its costs or contraindications. Symmetric substantia nigra hyperechogenicity coupled with frontal-horn dilatation points to early dementia with Lewy bodies, whereas an asymmetric signal and a low “Onset Index” favor Parkinson’s disease dementia. Vascular dementia, in turn, shows a sonographic triad of wide ventricles, sluggish middle-cerebral flow, and a stiff pulsatility profile, reflecting small-vessel disease.

Taken together, these findings position TCS as a rapid, bedside window onto serotonergic tone, nigrostriatal integrity, mesial-temporal atrophy, and cerebrovascular health. Its portability and low running costs make it especially attractive when MRI is impractical, and its real-time nature suits longitudinal follow-up in cognitive and psychiatric clinics. Standardized acquisition protocols and large, multicenter validation studies are now needed to translate these promising markers into routine personalized care.

## Introduction and background

Transcranial sonography (TCS) is broadly recognized as a valuable tool in the evaluation of movement disorders [1]. Nevertheless, it has also emerged as a pivotal imaging modality offering unique insights into brain structural alterations associated with a spectrum of neuropsychiatric disorders [2]. Among these, the hypoechogenicity of the brainstem raphe (BR) has garnered significant attention across diverse conditions such as major depressive disorder (MDD), bipolar disorder, and panic disorder. Research indicates a robust association between BR hypoechogenicity and clinical phenomena like depressive symptoms and MDD with suicidal ideation, further underscoring its predictive value for treatment response to serotonin reuptake inhibitors in MDD. Moreover, its potential contribution to the evaluation of dementia disorders has also been explored [3].

This article aims to review current literature on TCS applications beyond movement disorders, focusing on dementia and neuropsychiatric conditions, analyzing their diagnostic and prognostic potentials in clinical settings. By synthesizing findings across various disorders, it aims to summarize the current evidence regarding TCS in elucidating brain structural useful markers on diagnosis, follow‑up, treatment strategies, and prognostic assessments in neuropsychiatric and dementia issues. For a detailed review of TCS methods for image acquisition, see previous papers published elsewhere [4].

Methods

This is a narrative review that explores the utilization of TCS in the study of dementia and psychiatric conditions. The review focused on evaluating the TCS as a diagnostic and prognostic device in English‑language, peer‑reviewed journals.

This narrative review was conducted to explore the clinical applications of TCS in the evaluation of neuropsychiatric disorders, with a particular focus on dementia syndromes, including Alzheimer’s disease, as well as MDD, bipolar disorder, and anxiety disorders.

Relevant literature was searched in the PubMed, Scopus, and Web of Science databases. The search included peer-reviewed articles published in English up to January 2025, using combinations of keywords such as “transcranial sonography”, “transcranial ultrasound”, “dementia”, “Alzheimer’s disease”, "hippocampus", “depression”, "panic disorder", "anxiety", "obsessive‑compulsive disorder", "schizophrenia", “bipolar disorder”, and “psychiatric disorders”.

All retrieved articles were independently reviewed in full text by the authors. Studies were selected based on their methodological rigor, clinical relevance, and their contribution to understanding the diagnostic, prognostic, or pathophysiological role of TCS in the aforementioned conditions. Additional references were identified through manual screening of the bibliographies of included articles.

## Review

Discussion and results

TCS uses sectoral ultrasound probes with frequencies ranging from 1 to 5 MHz insonating through the acoustic temporal windows [4]. The routine axial and coronal planes of the method were discussed elsewhere [4]. When evaluating a patient with a possible dementia syndrome, the axial planes provide dimensions of the ventricular system and visualization of mesencephalic and basal nuclei. The coronal plane, orthogonal to the axial, depicts the mesial structures of the medial temporal lobe (MTL) [4].

TCS in neuropsychiatric disorders

Depression and Brainstem Raphe

**Figure 1:**
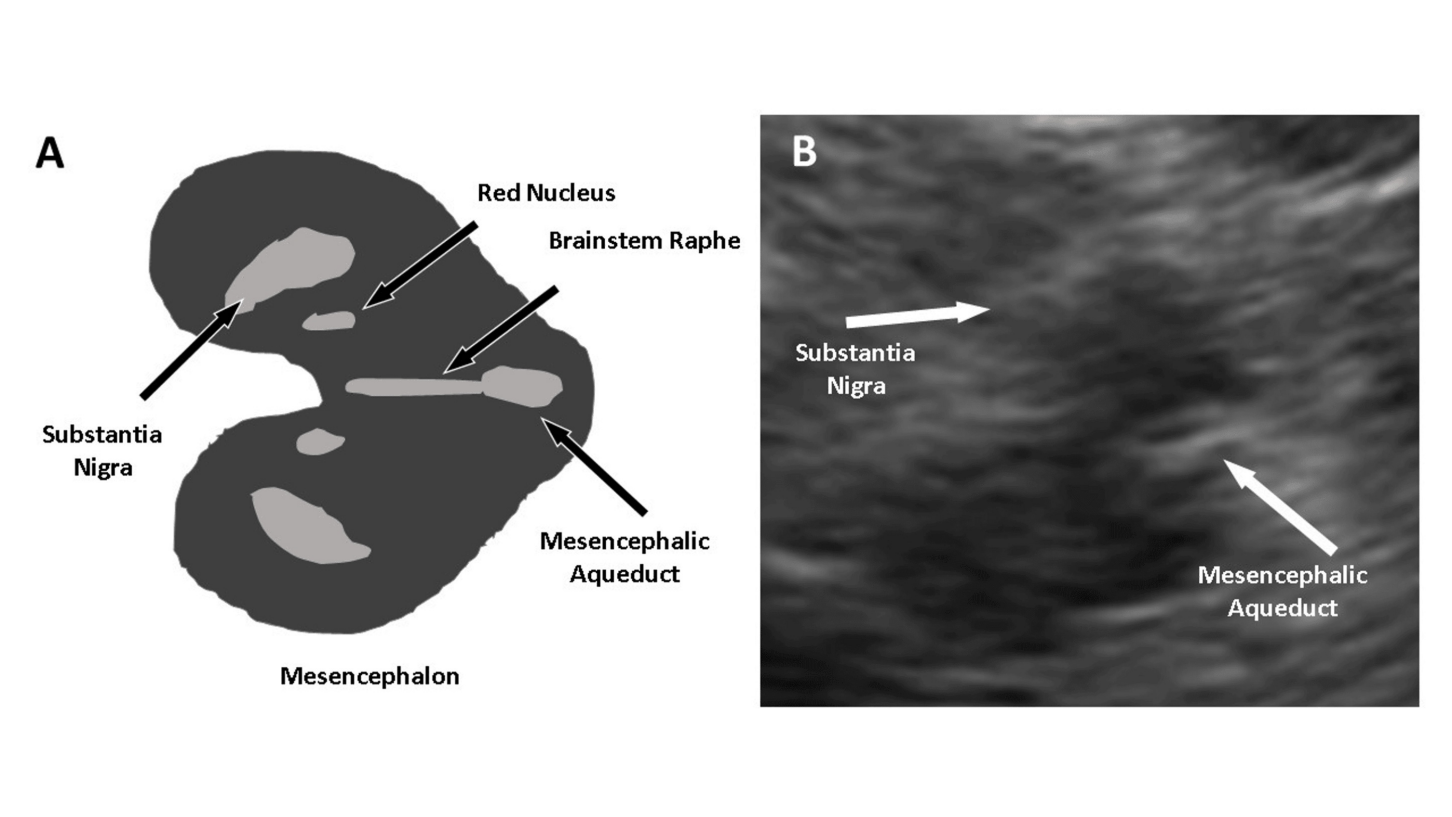
Mesencephalon view on axial plane. (A) Schematic representation of mesencephalic structures. (B) Transcranial sonography image of the mesencephalon. Image credit: Roberto P. Santos and Rita de Cássia L. Fernandes.

Major depressive disorder (MDD), along with suicidal ideation, has been associated with hypoechogenicity of the median raphe in transcranial sonography images (Figure 1) and is summarized in Table 1 [5-9]. On TCS, the brainstem raphe appears as a thin echogenic line bisecting the mesencephalon. In the normal state, the raphe’s signal intensity is similar to that of the basal cisterns and appears as a continuous line without interruptions. Echogenic abnormalities are classified as raphe hypoechogenicity when interruptions or absence of the raphe are observed from both insonation windows [4].

**Table 1:** Prevalence of brainstem raphe (BR) hypoechogenicity on transcranial sonography across neuropsychiatric and neurological conditions. Source: [4-8,10-23]. OR: odds ratio; MDD: major depressive disorder; SSRI: selective serotonin reuptake inhibitor; PPV: positive predictive value.

Condition/clinical context	Prevalence of BR hypoechogenicity	Control/comparator prevalence	Diagnostic/prognostic highlights
Major depressive disorder (all comers)	≈67%	10–15%	Predicts SSRI response (PPV ≈ 88%); correlates with episode count & severity
MDD with suicidal ideation	86%	–	Marker of suicidality risk
Drug-naïve or older-adult MDD	Up to 88%	–	Highest reported prevalence in untreated/elderly cohorts
Migraine (overall)	23–87%	–	Higher depression & anxiety scale scores; aura subtype linked to ↑ third ventricle width
Tension-type headache	13%	≈ controls	No clear association with BR echo changes
Bipolar disorder (all phases)	36%	–	Larger third ventricle width vs. controls; may reflect more severe mood phenotype
Panic disorder	64% (visual)/52% (digitized)	31%/12%	Sensitivity of 64%, specificity of 73% for panic disorder diagnosis
Obsessive–compulsive disorder	32%	16%	Data conflicting; not replicated in later studies
Post-ischemic stroke (3-month follow-up)	OR of 6.37 for new depression when BR is hypoechoic	–	Early prognostic marker for post-stroke depression

The hypoechoic BR can be observed in 47% of MDD patients, compared to 15% of controls. Among patients with suicidal ideation, this finding is even more common (86%) [5]. Similarly, other authors found BR hypoechogenicity in 66% of MDD patients [7]. The finding of abnormal, hypoechoic BR predicts good responsiveness to selective serotonin reuptake inhibitors (SSRIs), with a positive predictive value of 88% [7]. Among patients with Parkinson’s disease, this finding can be as frequent as 85% [8]. It has also been observed that homozygosity for the short allele of the serotonin transporter gene promoter region (5-HTTLPR), which is related to vulnerability to depression, was significantly higher in MDD patients with reduced BR echogenicity compared to those with normal BR echogenicity [7]. Additionally, MDD patients exhibiting motor symptoms and impaired verbal fluency were more likely to have both raphe hypoechogenicity and substantia nigra (SN) hyperechogenicity (see Fernandes & Santos (2025) [1] for a review of TCS in SN evaluation). These findings correlated with a higher incidence of MDD in those patients [9,10].

Some researchers have noted a reduction in BR echogenicity in approximately 67% of MDD patients, compared to only 10-15% of controls [5,6,10,11]. In patients with suicidal ideation, this prevalence reaches 86%. Moreover, raphe hypoechogenicity appears to correlate directly with the number of depressive episodes and the severity of the disorder [5,6]. Kostić et al. found raphe hypoechogenicity in 66% of MDD patients and in 9% of controls [7]. Studies including treatment-naïve patients and older adults tend to report even higher prevalence, around 88% [8,12].

Raphe hypoechogenicity has been reported in 23-87% of migraine patients, whereas tension-type headache patients show a rate (13%) similar to controls [8,13-16]. Those with raphe hypoechogenicity tend to score higher on depression and anxiety scales. Furthermore, migraine patients with comorbid MDD more frequently display raphe hypoechogenicity (83%) compared to those without MDD (0%) [15]. Migraine with aura is also associated with an increased third-ventricle diameter [14].

While no difference in BR echogenicity has been observed between manic and depressive phases, the severity of depression is linked specifically to the hypoechogenic subgroup, suggesting a broader association of BR hypoechogenicity with depressive symptoms. BR echogenicity may thus serve as a biological marker for depression or suicidality risk, aiding clinicians in assessment. Hypoechogenicity of the BR may also act as a prognostic marker: in the immediate post-ischemic stroke phase, patients with this finding had a higher incidence of depressive disorder at three-month follow-up compared to those with normal BR (48.0% vs. 4.1%, p < 0.001), even after adjustment for sex, modified Rankin scale (mRS) score, and previous depressive episodes (adjusted OR: 6.371) [17].

Depression and Substantia Nigra

In about 90% of Parkinson’s disease (PD) patients, TCS reveals characteristic SN hyperechogenicity [1]. This marked SN hyperechogenicity is also seen in approximately 10-15% of healthy adults aged 20-80 years and has been linked to nigrostriatal dopaminergic dysfunction in pharmacological, PET, and single photon emission computed tomography (SPECT) studies, suggesting an increased risk for PD development [18,19].

In depressive disorders, SN hyperechogenicity is observed with a three-fold higher frequency compared to non-depressed controls [18]. In their study of MDD and adjustment disorder with depressed mood (ADDM), Hoeppner et al. reported SN hyperechogenicity in 37% of patients and BR echogenicity in 54%. Frequency of SN hyperechogenicity did not differ between SSRI-treated and untreated patients (39% vs. 36%; p = 0.83), nor did BR echogenicity (48% vs. 57%; p = 0.47) (Table 2). Moreover, no correlation was found between SN echogenic size and depression severity [18].

**Table 2:** Frequency of substantia nigra (SN) hyperechogenicity on transcranial sonography across neurological and psychiatric cohorts. Source: [1,11,18,19]. ADDM: adjustment disorder with depressed mood; BR: brainstem raphe; DIP: drug-induced parkinsonism; MDD: major depressive disorder; PD: Parkinson’s disease; TCS: transcranial sonography; SSRI: selective serotonin reuptake inhibitor.

Condition	% with SN hyperechogenicity	Notes
Parkinson’s disease	≈90%	Classic TCS hallmark of PD
Drug-naïve healthy adults	10-15%	Potential PD risk marker
Major depressive disorder/ADDM	37%	3-fold increase vs. controls; unrelated to SSRI use or depression severity
Bipolar disorder	16.7%	Small cohort; co-occurs with BR hypo-echo (36%)
Schizophrenia + antipsychotics (drug-induced parkinsonism)	20-30% overall; higher in persistent cases	Larger SN area ⇢ more severe parkinsonian signs

Bipolar Disorder: Brainstem Raphe and Hippocampus

Accelerated hippocampal atrophy, whether due to chronic illness progression or medication effects, has been documented in bipolar disorder. Lower hippocampal volumes on MRI and enlarged lateral ventricles have been reported in these patients [20]. To our knowledge, only Becker et al. [2] and Krogias et al. [11] have investigated bipolar disorder using transcranial sonography. Consistent large-scale MRI findings support the plausibility of detecting similar structural changes by TCS and evaluating their diagnostic and prognostic value. Krogias et al. found that 16.7% of participants exhibited SN hyperechogenicity, while 36.1% showed BR hypoechogenicity (Table 1) [11]. Among bipolar patients in a depressive episode, BR echogenicity was notably reduced on TCS. They also observed a significantly larger third-ventricle width in patients compared to controls (3.8 ± 2.1 mm vs. 2.7 ± 1.2 mm). Although the differences in altered echogenicity frequencies were not statistically significant, likely due to the small sample size (36 cases: 14 depressed, 8 manic, 14 euthymic; 35 controls), these results suggest that BR hypoechogenicity may reflect greater clinical severity and is not specific to unipolar depression [11].

Panic Disorder and Brainstem Raphe

Mesencephalic raphe hypoechogenicity has also been documented in panic disorder patients versus healthy controls, both by visual assessment (68% vs. 31%) and digitized image analysis (52% vs. 12%). A hypoechoic raphe on TCS demonstrated 64% sensitivity and 73% specificity for diagnosing panic disorder [21].

Obsessive-Compulsive Disorder (OCD) and TCS

Early TCS studies reported a higher prevalence of raphe hypoechogenicity in OCD patients compared to controls (32% vs. 16%) and increased caudate-nucleus hyperechogenicity (31% vs. 6.5%) (Table 1) [22]. However, subsequent investigations failed to replicate these findings [23], indicating that further research is needed to clarify the relationship between TCS-detected echogenic changes and OCD.

Schizophrenia and Substantia Nigra

Antipsychotic-induced parkinsonism in schizophrenia poses a diagnostic challenge. In idiopathic PD, about 90% of patients show SN hyperechogenicity on TCS; in secondary parkinsonisms, this finding is present in only 20-30% (Table 2) [1]. Thus, SN hyperechogenicity has a negative predictive value of 78-87% for ruling out secondary parkinsonism [24]. Nonetheless, some patients initially diagnosed with drug-induced parkinsonism do exhibit SN hyperechogenicity [25], suggesting a latent dopaminergic degeneration unmasked by medication [26]. This aligns with clinical data showing that ~20% of patients with medication-induced parkinsonism continue to worsen despite drug withdrawal [27,28]. Moreover, larger SN hyperechogenic areas correlate with more severe Parkinsonian signs [27].

TCS and dementia

Dementia is increasingly prevalent as populations age, posing a major public health challenge [29]. Defined as acquired cognitive decline severe enough to impair social or occupational function [29], dementia is most often driven by Alzheimer’s pathology and cerebrovascular disease [30,31]. Brain-parenchymal atrophy in dementia affects gray and white matter variably across regions [29]. TCS has been applied to evaluate ventricular size, mesencephalon, and MTL structures in these syndromes.

Several studies report larger third-ventricle diameters on TCS in cortical dementias [32-34] and Parkinson’s disease dementia (PDD) [35]. Third-ventricle width inversely correlates with Mini-Mental State Examination (MMSE) and Montreal Cognitive Assessment (MoCA) scores [32]. In a five-year longitudinal study of 500 healthy elders, Wollenweber et al. observed universal ventricular enlargement with aging, but those with marked cognitive decline had significantly greater enlargement [36]. These data underscore the potential of TCS ventricular measurements as noninvasive markers of brain atrophy [32-34,36].

TCS in Alzheimer’s Disease (AD)

**Figure 2:**
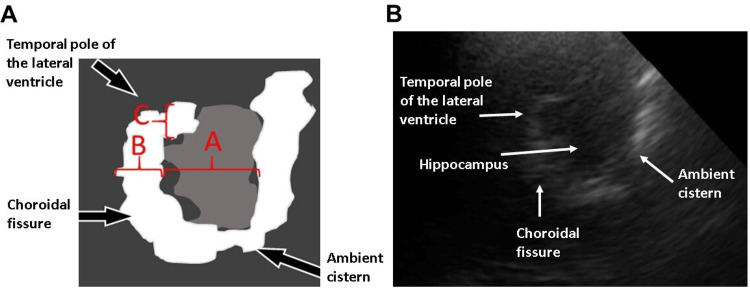
Medial temporal lobe (MTL). (A) Schematic picture of MTL and references of measures of its structures. A - Height of the hippocampus. B - Height of the choroidal fissure. C - Width of the temporal pole of the lateral ventricle. (B) Sonographic image of MTL and its structures. Figure credit: Roberto P. Santos and Rita de Cássia L. Fernandes.

Two key studies assessed MTL structures by TCS in AD [37,38]. In 2016, authors demonstrated that the ratio of MTL height to choroidal-fissure height (Figure 2) discriminated AD patients from controls with 83% sensitivity and 76% specificity at a cutoff of < 2.4 (Table 3), comparable to MRI accuracy [37]. For detailed TCS techniques and normal intracranial-structure values, see Fernandes & Santos (2025) [4].

**Table 3:** Assessment of the mesial temporal lobe by transcranial sonography for the diagnosis of Alzheimer’s disease. MTA: medial temporal lobe atrophy; MTA-S: MTA score in sonography; VES-S: ventricle enlargement score in sonography = right temporal pole height (coronal plane) + left temporal pole height (coronal plane) + internal diameter of the third ventricle; Ratio M/F: ratio of the height of the mesial temporal lobe/height of the choroidal fissure [37-39].

Measure	Sensitivity	Specificity
MTA-S ≥ 8.4 mm	84%	83%
VES-S ≥ 15 mm	84%	93%
Ratio M/F ≤ 2.4	73-83%	72-76%

In a second published study, validation of TCS measurements was performed against the gold‐standard MRI mesial temporal lobe atrophy (MTA) scale [38]. The authors proposed a sonographic atrophy score and demonstrated a strong correlation between MTA score in sonography (MTA-S) and MRI-based MTA. The MTA-S is calculated as the sum of the temporal‐pole height of the lateral ventricle and the choroidal fissure height (Figure 2). An MTA-S < 8.4 mm differentiates AD patients from healthy controls with 84% sensitivity and 83% specificity. An MTA-S < 6 mm corresponds to no MTL atrophy (equivalent to MTA grade 0-1 on MRI). Conversely, an MTA-S > 10 mm indicates significant atrophy, corresponding to MTA grade > 2 on MRI [39].

Another approach to assessing MTL atrophy by TCS is the ventricle enlargement scale in sonography (VES-S), calculated as the sum of the third-ventricle width plus the right and left temporal pole widths. A VES-S > 15 mm is highly suggestive of AD [38]. Further studies are needed to confirm these findings and establish TCS as a reliable alternative for patients with MRI contraindications (e.g., metallic prostheses, pacemakers, or claustrophobia).

TCS in Parkinson’s Disease Dementia (PDD) and Dementia With Lewy Bodies (DLB)

PDD frequently develops in patients with longstanding PD. Unlike AD, it predominantly impairs executive function and attention, with relatively preserved language, memory, and calculation abilities [40]. TCS demonstrates progressive third-ventricle enlargement in PD patients with cognitive decline [41], and reduced mesencephalic area, especially in those with subcortical dementia syndromes such as progressive supranuclear palsy [42,43].

When dementia precedes, or emerges within one year of Parkinsonism, it most likely reflects cortical α-synucleinopathy (DLB) [40]. These patients often have diurnal fluctuations and visual hallucinations. MRI may show posterior-parietal atrophy with relative hippocampal preservation [35,40]. TCS in DLB reveals SN hyperechogenicity (SN+) in approximately 80% of cases, similar to PD, implying shared pathogenesis [35].

In Favaretto et al.'s (2016) study [44], TCS of the SN differentiated DLB from AD: 100% of DLB patients showed SN+, with > 50% having marked hyperechogenicity (area > 0.22 cm²), versus 50% of AD patients and 30% of controls. Mean SN area was larger in DLB (0.22 ± 0.03 cm²) than in AD (0.15 ± 0.03 cm²) and controls (0.14 ± 0.03 cm²) (p < 0.0001). SN hyperechogenicity in DLB was predominantly symmetrical, whereas AD exhibited greater asymmetry (asymmetry index (AI) > 1.15 in 51.8% vs. 18.2%; p = 0.015). Combining enlarged SN area with low asymmetry (AI < 1.15) yielded sensitivity of 88.9%, specificity of 81.8%, and predictive values of 85.7% for DLB [43]. Clinically, DLB patients had higher rates of rigidity (72.7% vs. 25%; p < 0.001), visual hallucinations (63.6% vs. 7.1%), and rapid eye movement (REM) sleep behavior disorder (78.2% vs. 14.3%) than AD, and SN+ did not correlate with Unified Parkinson's Disease Rating Scale III (UPDRS-III) or MMSE scores, supporting its role as a susceptibility marker rather than a severity index [43].

In a cohort with ≤ three years of symptoms, bilateral SN+ (area ≥ 0.20 cm²) was found in 50% of DLB versus 27% of PD, strengthening the suspicion of DLB when present. Frontal-horn dilatation further increased DLB odds versus PD (ORs ≈ 9.5 and 5.7; p ≈ 0.05). The combination of SN+ and frontal-horn enlargement provided maximal diagnostic leverage in early, ambiguous cases [44].

Another index, the “Onset Index,” incorporates patient age at symptom onset and the sum of both SN areas, divided by the asymmetry index. An onset index > 35.5 suggests DLB over PDD (Table 4) [1,35].

**Table 4:** TCS accuracy in the differential diagnosis between Lewy body dementia and Parkinson's disease dementia. Adapted from Fernandes and Santos (2025) [1] and Walter et al. (2006) [43]. Asymmetry index = (larger SN ÷ smaller SN); Onset index = {(age of disease onset x sum of echogenic area of bilateral SN) ÷ asymmetry index} [1,35,43,44]. TCS: transcranial sonography; LBD: Lewy body dementia; SN: substantia nigra; PDD: Parkinson's disease dementia.

TCS marker/cut-off	Indicated condition	Main differential excluded	Sensitivity	Specificity
Asymmetry index ≥ 1.15	PDD	LBD	69%	80%
Onset index > 35.5	LBD	PDD	96%	80%

TCS in Vascular Dementia

Vascular dementia (VaD) is a major public health concern as the second most frequent dementia after AD, and it often coexists with Alzheimer’s pathology [45]. Historically termed multi‐infarct dementia, VaD reflects cumulative tissue loss from recurrent strokes. Various mechanisms, including large‐ and small‐vessel disease and hemorrhagic strokes, are encompassed under the more inclusive designation “vascular cognitive impairment” [45].

Neurosonology, comprising transcranial duplex (Doppler and B-mode) and extracranial ultrasound (carotid and vertebral arteries), is a noninvasive, widely available, and repeatable method that permits real‐time hemodynamic assessment of cerebral arterial flow, alongside measurement of ventricular diameters [46]. Reduced mean flow velocities and decreased intracranial vascular resistance on Doppler suggest hypoperfusion secondary to cerebral arterial atherosclerosis in patients with white-matter lesions and vascular cognitive impairment [47,48].

Multiple studies have documented significant neurosonographic differences between VaD patients and age-matched AD patients or healthy controls. VaD patients typically exhibit lower mean velocities and higher pulsatility indices (PI) in the middle cerebral arteries compared to AD patients, as well as impaired cerebrovascular reactivity [49-52]. These findings likely reflect increased peripheral resistance due to small-vessel stiffness in VaD [49].

The role of vascular factors in neurodegenerative disease onset and progression has only recently become a focus of research. A growing number of investigations demonstrate correlations between higher Fazekas scores on cerebral MRI and impaired cerebrovascular reactivity on TCS in PD and AD cohorts [53-56]. Small-vessel disease’s contribution to neurodegeneration remains debated, but TCS offers a safe means of assessing cerebral hemodynamics in these contexts.

Going forward, combined Doppler and B-mode ultrasound could deliver real-time functional mapping of the cerebrovascular network and parenchymal structures at relatively low cost. The triad of enlarged ventricles, reduced flow velocities with elevated pulsatility, and diminished cerebrovascular reactivity is highly suggestive of VaD. Thus, TCS is evolving not only as an acute ischemia tool but also as a potential preclinical marker of vascular pathology.

Limitations of the study

Although TCS represents a promising tool for the assessment and monitoring of various conditions beyond movement disorders, there are several limitations to consider. Firstly, the interpretation of TCS findings can vary significantly due to the still poor standardization in imaging acquisition and analysis methods, limited access to trained personnel, lack of reimbursement structures, and institutional unfamiliarity with the method. Additionally, image quality may be affected by challenges in obtaining clear and precise images, such as the individual temporal bone window. Furthermore, the clinical applicability of TCS may be restricted in patients with atypical anatomical features or conditions that hinder adequate visualization of brain structures.

These limitations are further exacerbated by the very limited number of studies currently available, most of which are small in scale and methodologically heterogeneous. The current body of evidence remains limited and preliminary. This highlights the need for further, larger, and more standardized studies to investigate the potential clinical utility of TCS as a diagnostic tool in neuropsychiatric and dementia disorders.

Future perspectives

The applicability of TCS in dementia and neuropsychiatric disorders remains to be fully established, and further evidence is needed to determine its clinical utility in these fields. Ongoing technological advancements have led to ultrasound devices with progressively higher resolution, enabling clinicians to non-invasively assess brain structures involved in various psychiatric disorders with increasing precision and reliability. Moving forward, there is a growing interest in expanding the utility of TCS beyond traditional diagnostic roles to include therapeutic monitoring and treatment guidance. Moreover, ongoing research efforts aim to establish standardized protocols and refine imaging techniques, which might enhance the clinical applicability and utility of TCS in psychiatric and dementia approaches.

## Conclusions

TCS represents a rapid, radiation-free, and cost-effective neuroimaging modality capable of visualizing deep brain structures at the bedside. Its image resolution remains significantly lower compared to other modalities such as MRI. Nonetheless, TCS may serve as a complementary imaging method in specific clinical contexts. Its portability and absence of ionizing exposure render it particularly advantageous in resource-limited settings or in patients for whom computed tomography or MRI are contraindicated. Recent technical refinements have enhanced image resolution and reproducibility, positioning TCS as a credible alternative or complementary tool for routine intracranial assessment.

Despite its promising clinical applications, the current evidence supporting the use of TCS as a neuroimaging biomarker in neuropsychiatric and neurodegenerative disorders remains limited. Most available studies are preliminary in nature, with small sample sizes and significant methodological heterogeneity. These limitations hinder the generalizability of findings and underscore the need for robust, large-scale studies aimed at standardizing and validating this imaging modality before its widespread incorporation into clinical practice. The existing knowledge gaps and the preliminary nature of current findings encourage further research to explore the role of TCS in these clinical conditions. Continued investigation may help clarify its potential utility in the diagnostic approach to dementia and neuropsychiatric disorders.
